# Dynamic Simulation of a Fe-Ga Energy Harvester Prototype Through a Preisach-Type Hysteresis Model

**DOI:** 10.3390/ma12203384

**Published:** 2019-10-17

**Authors:** Stefano Palumbo, Mario Chiampi, Oriano Bottauscio, Mauro Zucca

**Affiliations:** 1 Istituto Nazionale di Ricerca Metrologica, INRIM, Strada delle Cacce 91, 10135 Torino, Italy; s.palumbo@inrim.it (S.P.); m.chiampi@inrim.it (M.C.); o.bottauscio@inrim.it (O.B.); 2 Politecnico di Torino, Department DET, Corso Duca degli Abruzzi 24, 10129 Torino, Italy; 3 Istituto Italiano di Tecnologia, IIT, Graphene Labs, Via Morego 30, 16163 Genova, Italy

**Keywords:** energy harvesting, finite element model, iron–gallium, measurements, preisach model

## Abstract

This paper presents the modeling of an Fe–Ga energy harvester prototype, within a large range of values of operating parameters (mechanical preload, amplitude and frequency of dynamic load, electric load resistance). The simulations, based on a hysteretic Preisach-type model, employ a voltage-driven finite element formulation using the fixed-point technique, to handle the material nonlinearities. Due to the magneto–mechanical characteristics of Fe–Ga, a preliminary tuning must be performed for each preload to individualize the fixed point constant, to ensure a good convergence of the method. This paper demonstrates how this approach leads to good results for the Fe–Ga prototype. The relative discrepancies between experimental and computational values of the output power remain lower than 5% in the entire range of operating parameters considered.

## 1. Introduction

There has long been interest in giant magnetostrictive materials (GMMs). The first real application of a magnetostrictive material for actuators with marine sonar dates to the late 1970s. In the mid-1980s, Terfenol-D became commercially available and new high-energy-density devices were developed, along with design tools suitable for predicting the dynamic performance of GMMs. In the 1990s, the first attempts to model GMMs, in which the material is simply considered as nonlinear, were proposed [1,2,3,4,5]. In more detail, the model was considered 1D [2], or the magneto–elastic coupling was neglected [4,5].

In the 2000s, along with an improvement in the devices, came the development of more accurate general mathematical models of magnetostrictive materials [6,7]. The piezomagnetic equations were presented in early papers, such as [7]. Even though these early papers were only focused on models of the magnetostrictive material, new approaches showed the coupling of electromagnetic and magnetoelastic phenomena, together with the development of advanced hysteresis models [8,9,10,11,12]. Finally, the Armstrong model-based approach was applied, to simulate an entire device [13], in which the coupling variables were magnetostriction and magnetic permeability. A more general approach was based on hysteretic energy-weighted models [14], where magnetic field and stress were assumed as the state variables. Other approaches, based on phenomenological models of magnetostriction, adopted genetic algorithms [15,16] or used classical Preisach operators and a memoryless bivariate function, including a pure elastic contribution [17].

In the last decade, interest has shifted from actuators to energy-harvesting devices. An approach based on a Preisach-type magnetoelastic model was proposed by the authors of [18], for the analysis of giant magnetostrictive actuators based on Terfenol-D. From this, the authors proposed a magneto–mechanical (MM) hysteresis model of the magnetostrictive material [19], in which the Preisach model is modified, introducing an effective field that is the sum of the applied field, and a corrector term, which is a function of the mechanical stress. Such a model was employed for the design of an energy harvester based on a Terfenol-D rod.

Despite its larger magnetostriction (*λ_s_* ~1200–1600 ppm), Terfenol-D has been gradually surpassed by Fe–Ga alloys (*λ_s_* ~250–350 ppm). The reasons for this overtaking, which are analysed in [20], can be briefly summarized as follows: high tensile strength, ductile nature, and low hysteresis losses, combined with strong magneto–elastic coupling. In addition, Fe–Ga alloys show steel-like structural properties, and can be machined easily, either welded or rolled.

Several approaches to Fe–Ga alloy modelling can be found in the literature, differing in the respective constitutive equations they are based on. A static finite element method (FEM) approach, based on the Armstrong model and presented in [21], is characterized by a so-called weak coupling between the magnetic and mechanical problem. A phenomenological model, proposed in [22], considers the effects of the hysteresis phenomena on the mechanical and magnetic energy exchanges, and is consistent with classical non-equilibrium thermodynamics. An improved Preisach-based phenomenological model is the core of [23]. Here, emphasis was placed on the effect of hysteresis, such as energy dissipation and non-differentiability of the Preisach operator. The non-hysteretic model in [24] is based on the Gibbs free energy, and characterised by a realistic magneto–mechanical modelling. The paper is unusual in its representation of the mechanical, magnetic, and electrical parts of the device in a nonlinear three-port circuit. In [25], an interesting comparison between three mathematical models is reported, one based on the Preisach operator and the other two on a non-hysteretic model, with or without a feedback loop. Finally, a thermodynamic approach based on Helmholtz free energy density is presented in [26], where the magnetic flux density and mechanical strain are assumed as state variables. This choice leads to a simplified implementation of FEM models, avoiding the problem of constitutive law inversion, which is time-consuming.

In this paper, drawing from the model developed in [19], a specific identification has been carried out for a Fe–Ga rod, in order to reproduce its non-linear magnetic characteristics, starting from a set of experimental curves. Then, the behaviour of a harvester generator prototype, coupled to an electric circuit with a load resistance, has been simulated in dynamic conditions through a voltage-driven finite element model. Particular attention is devoted to the convergence of the fixed point (FP) method, adopted to handle the Fe–Ga non-linearity. Since the convergence is strongly affected by the preload, a preliminary tuning of the correct FP constant is adopted, to significantly improve the accuracy of the device output quantities (voltage and power). The proposed approach has been tested in a large range of values of operating parameters (mechanical preload, frequency and amplitude of the dynamic mechanical load, electric load resistance). The comparison of the measurements with a harvester prototype, specifically realized with the simulation results, show a more than satisfactory agreement.

## 2. Materials and Methods 

**Figure 1:**
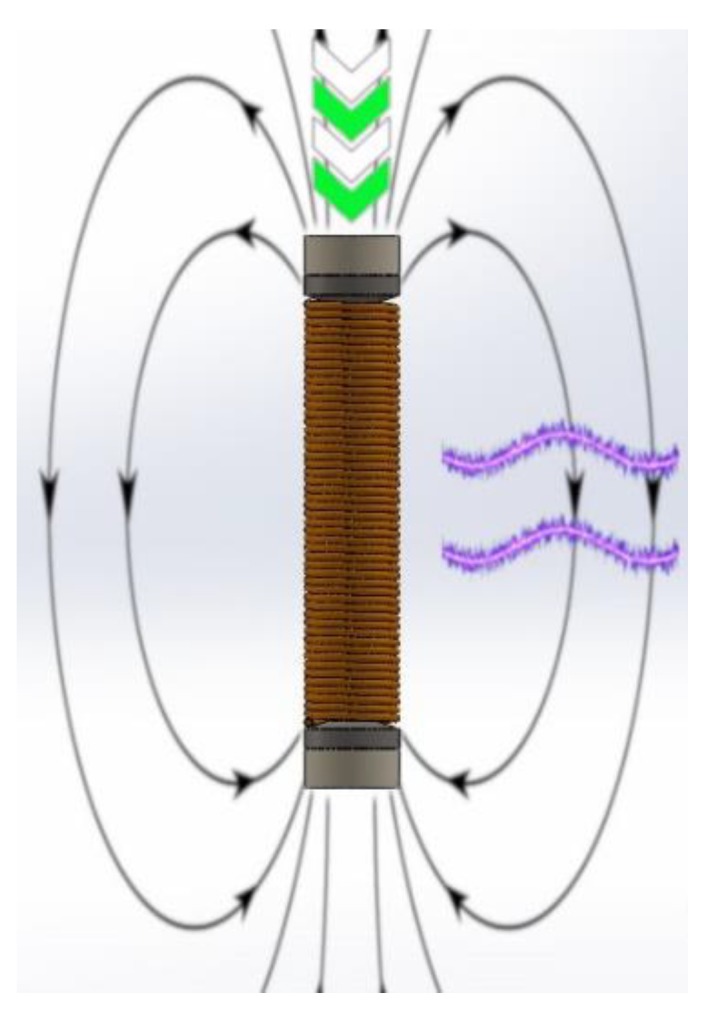
Schematic representation of the magnetostrictive principle. By applying a dynamic stress on an Fe–Ga rod within a mechanical preload and a magnetic bias, it is possible to generate a sinusoidal current inside the pick-up coil.

The principle of the magnetostrictive generator is well known: in the presence of a mechanical preload and a magnetic bias, a dynamic force is applied to a Fe–Ga rod, usually cylindrical. The applied stress dynamically modifies the magnetic permeability of the magnetostrictive material, giving rise to time variations of the magnetic flux flowing in the rod, so that an electromotive force is induced in a pick-up coil wrapped around the rod. The coil is connected to an electric circuit, where the circulating current transfers power to a suitable load. A scheme of the device is presented in Figure 1.

In the generator, the independent quantities are the mechanical preload $\sigma_{0}$, the amplitude and frequency of the dynamic applied stress *σ(t)* and the magnetic bias, and these are usually produced by permanent magnets chosen during the design phase. The output are the electrical quantities (voltage and current), or simply the electric power transferred to the load.

### 2.1. Magneto-Mechanical Preisach Model of the Fe–Ga Rod

Magnetostrictive materials are characterized by a strong link between magnetic and mechanical properties, both characterized by hysteresis. A good model of this complex behaviour is essential for an accurate simulation of a magnetostrictive device.

The present work adopts a modified Preisach model, described in [27], which is based on the introduction of an effective field, *H_e_*. The latter is the sum of the applied external field *H* and a corrector term *ξ(J,σ)* [28,29], which, in turn, is a function of the magnetic polarization *J* and of the mechanical stress *σ*:

(1) $$H_{e}=H+\xi (J,\sigma )$$

The magnetic flux density *B* is expressed as:

(2) $$B=\Psi \left(H\right)=\mu_{0}H+J=\mu_{0}H+\varsigma \left(H_{e}\right)$$

where the magnetic polarization *J* is a function of *H_e_* and includes both reversible and irreversible contributions. The hysteresis contribution is introduced by the function ς, described through the Classical Preisach Model (CPM). In Equations (1) and (2), and in the following relations, dependence on time is omitted from all field quantities for the sake of simplicity.

The effective field *H_e_* introduces a correction in the MS-Preisach model that makes it implicit. The nonlinear relation Ψ between *B* and *H* is handled by the Fixed Point (FP) technique, which splits the nonlinearity into a linear term with constant coefficient *η* and a residual **R**, to be iteratively updated. The iterative numerical scheme is described in detail in [18].

### 2.2. Finite Element Model of the Generator

The equations, which govern the dynamic behaviour of the generator, involve both the electromagnetic field and the electric circuit connected to the pick-up coil, requiring the adoption of a voltage-driven formulation able to simultaneously solve the two sets of equations.

Drawing on the harvester geometry, the device is described in a cylindrical reference system of coordinates *r, z, ϑ*, leading to a 2D axis-symmetric problem. The electromagnetic field is developed in terms of a magnetic vector potential **A** (*B* = *curl***A**), with only the *ϑ*-component, whose distribution is the unknown in the electromagnetic problem.

Having applied the FP technique, the electromagnetic field equation in the weak form becomes:

(3) $$\int_{\Omega }\nu curlA\cdot curlwds=-\int_{\Omega }R\cdot curlwds+\int_{\Omega_{c}}\frac{N_{c}i_{c}}{S_{c}}wds-\frac{1}{\rho }\int_{\Omega_{r}}\dot{A}wds+\frac{1}{\rho }\int_{\Omega_{r}}M_{\Omega_{r}}\left(\dot{A}\right)wds$$

where **w** is the test function, *ν* is the magnetic permittivity (*η* in the magnetostrictive rod and vacuum value elsewhere), **R** is the FP residual and Ω is the 2D full domain under study. Subdomain Ω*_r_* is the trace on Ω of the rod with electrical conductivity *ρ*, and **M**_Ω_ denotes the mean time derivative of **A** over Ω*_r_*. Subdomain Ω*_c_* is the trace (with area *S_c_*) on Ω of the pick-up coil, which has *N_c_* turns where the unknown current *i_c_* is flowing.

The problem is completed by the electric loop equation of the pick-up coil:

(4) $$R_{l}i_{c}+L_{l}\frac{di_{c}}{dt}+e_{c}+R_{c}i_{c}=0$$

where *R_c_* is coil resistance (*R_c_* = 32.6 Ω), *R_l_* and *L_l_* are resistance and stray inductance of the electric load and the electromotive force induced at the coil terminals is expressed in terms of **A**, as:

(5) $$e_{c}=\frac{2\pi N_{c}}{S_{c}}\int_{\Omega_{c}}r\dot{A}ds$$

The field equation is solved through the Finite Element Method (FEM), suited for the analysis of these kind of devices (see, for example, [30,31,32]). The domain Ω is suitably enlarged, to allow homogeneous Dirichlet conditions on its boundaries, and discretized using first-order elements. The time evolution is handled by adopting a step-by-step scheme.

From a mechanical point of view, the mechanical force at each time step is assumed to be applied normally to the rod cross-section with only the axial-component. Taking advantage of the cylindrical geometry, the applied stress can be considered uniformly distributed at each point of the rod and computed simply as the ratio of the instantaneous force to the rod cross-section. This is a simplification, which avoids the solution of a mechanical problem by the finite element method to compute the spatial distribution of stress within the rod. It has been verified in other studies [18], related to magnetostrictive actuators, that this hypothesis is acceptable. The elongation of the magnetostrictive rod, defined as Δ*L* = Γ*(σ,J)L*, is computed by averaging the local strain*λ* over a rod cross-section Σ_r_ and length *L*:

(6) $$\Gamma =\frac{2\pi }{V_{r}}\int_{\Sigma_{r}}\lambda rds$$

where *V_r_* is the volume of the rod.

However, the limited size of the strain inside the rod (a few hundred ppm) does not justify a remeshing of the FEM domain during the solution of the electromagnetic field problem. Therefore, it is assumed that the rod shape is remains unchanged throughout the entire computational process. All computations required for the device simulation are performed using a homemade code (*Sally2D*), which is able to manage hysteretic materials.

### 2.3. Magnetostrictive Properties

**Figure 2:**
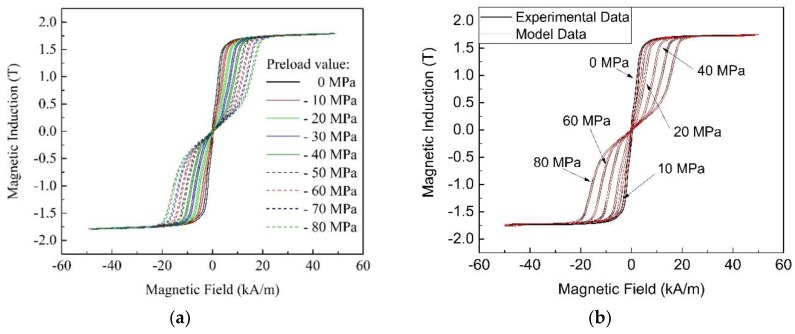
(**a**) Measured quasistatic B–H loops for different levels of the constant preload; (**b**) superposition of model data B–H loops on experimental B–H loops.

The Preisach distribution Φ*(α,β)*, together with the corrective function *ξ* (used in Equation (1)), and the strain function *λ* (as appears in Equation (6)), are necessary to fully identify the MS-model. These functions must be experimentally determined, starting with Fe–Ga material characterization. The procedure for quasi-static material characterization, described in [10], includes the measurement of magnetic hysteresis loops (see Figure 2a) and rod elongation at different mechanical loads.

Starting from an experimental loop (*H_exp_*, *B_exp_*), the simulated effective field *H*_e_ is computed and then compared (see Figure 2b) with the experimental waveform:

(7) $$\begin{array}{l} J_{exp}=B_{exp}-\mu_{0}H_{exp}\\ H_{e}=\zeta^{-1}(J_{exp})\\ \xi (J_{exp},\sigma_{exp})=H_{e}-H_{exp} \end{array}$$

where the inversion of the Preisach model (ζ^−1^) is performed through a numerical procedure. The application of this procedure provides a set of discretized values of the experimental curves of *ξ* and *λ* in the plane *J, σ.* Since, unlike Terfenol-D, analytical functions able to interpolate the curves in the whole plane cannot be determined, the simulated data must be reconstructed through a four-point spatial interpolation. 

This approach, based on interpolation, without the possibility of reasonably extrapolating data, limits the maximum stress applicable, since the sum of mechanical preload and dynamic load cannot overcome the highest static load applied during the experimental material characterisation (in this work, 80 MPa).

### 2.4. Harvester Prototype Setup and Modeling

**Figure 3:**
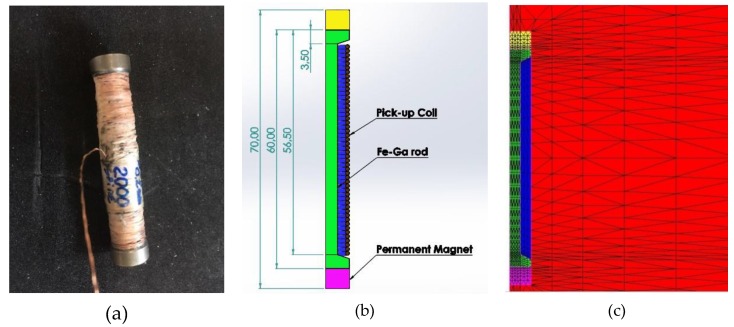
EH system: (**a**) Actual picture of the Fe–Ga rod with a pick-up coil of 2000 turns and a resistance of 32.6 Ω; (**b**) Sketch of the rod aligned section with permanent magnets and pick-up coil. Dimension in mm; (**c**) 2D mesh for a limited portion of the computational domain Ω used for the FEM analysis (Fe–Ga in green, pick-up coil in blue, PMs in yellow and purple, air in red).

The direct force harvester is based on the use of a polycrystalline Fe_81_Ga_19_ sample (cubic grains with <100> easy axes) in the shape of a cylindrical rod, with a length of 60 mm and a diameter of 12 mm. Thanks to the material workability, the rod was machined, reducing the external diameter to 6 mm from a length of 53 mm, in order to host a 2000-turns pick-up coil with 32.6 Ω electric resistance. The magnetic bias was generated by a couple of permanent magnets (PMs) placed at the extremities of the rod. A picture of the system, including the rod with the pick-up coil coated with white Teflon, is shown in Figure 3a, while a scheme is presented in Figure 3b.

The axis-symmetric structure of the prototype is discretized into triangular finite elements. A portion of the considered domain Ω is shown in Figure 3c, where each colour indicates a different material: air (red), Fe–Ga (green), pick-up coil (blue), PMs (yellow and purple). 

### 2.5. Characterization and Experimental Setups

The setup for the experimental characterization of the magnetostrictive material is based on a three-legged magnetizer and a test machine, and provides the hysteresis loops of Figure 2a, as described in [33,34]. 

**Figure 4:**
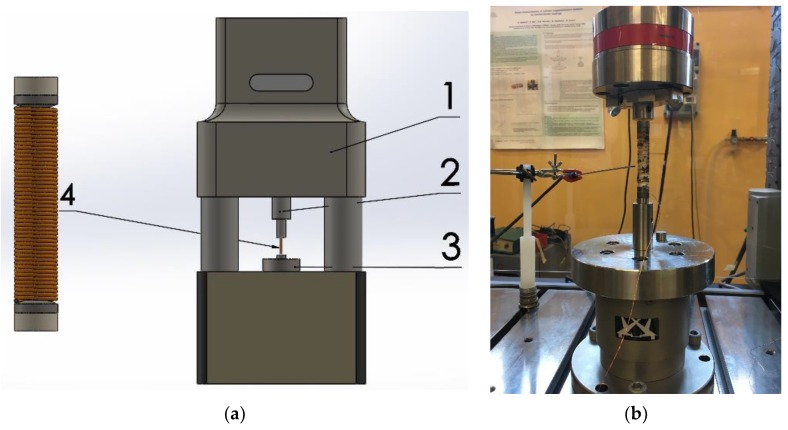
(**a**) 1. Fatigue testing machine applying the mechanical load through 2. plunger; using a 3. load cell for the feedback loop. The magnetic field bias is applied to the Fe–Ga rod 4. through a pair of PMs; **(b**) Picture of the Fe–Ga rod, with PMs inserted in the fatigue-testing machine for experimental analysis.

The experimental setup for the measurements on the Fe–Ga direct-force harvester (Figure 4) makes use of many of the experimental components described in [33]. In particular, a 10 kN-100 Hz fatigue-testing machine and a control software have been used to generate fully controlled harmonic vibrations. A programmable resistor (Pickering PXI 40-297-002 programmable precision Resistors, Pickering Interfaces Ltd., Clacton-on-Sea, Essex, UK) works as an electric load. Output power is measured, along with other electrical parameters, by a wattmeter Yokogawa WT 3000 (Yokogawa Electric Co., Musashino, Tokyo, Japan) using a 2-ampere channel, with a minimum range of 5 mA.

## 3. Results

### 3.1. Preliminary Results

In the experimental setup, the magnetic bias was provided by two Nd–Fe–B PMs with 955 kA/m coercive field and 1.2 T remanence and kept constant during all measurements. The load resistance was set to 160 Ω (near the peak output power), to be added to the 32.6 Ω coil resistance. The vibration force magnitude and the mechanical bias can be modified. However, in preliminary comparisons between experiments and numerical predictions, the dynamic vibration force peak was set to 8 MPa, with a fixed frequency of 100 Hz. Thus, in the preliminary tests, the mechanical preload $\sigma_{0}$ was the only parameter left free. This choice comes from the experimental analysis carried out in [34], where it was highlighted that, for a given magnetic field bias, only one value of the mechanical bias is able to maximize the output power. The mechanical preload $\sigma_{0}$ ranges from 30 MPa to 70 MPa, since the dynamic load is kept to 8 MPa.

As stated previously, the FEM model adopts the FP technique as an iterative method to handle the nonlinearities. This implies the use of a suitable FP constant *η,* and of a limit value of the convergence index together with the maximum number of iterations. 

The value of *η* is an essential parameter, because it affects both the convergence of the iterative method and its speed. In the presence of invariable magnetic characteristics (or quite close to them, as for Terfenol-D), the optimal value of the FP constant is chosen, starting from the maximum and minimum slopes of the B-H curve. The average value between the two slopes guarantees a regular convergence of the iterative process. However, in the Fe–Ga alloy, the magnetic behavior significantly varies with the mechanical load (sum of the preload and dynamic load), as illustrated by the curves in Figure 2a, which show an evident bending at the preload increase. In addition, the presence of a magnetic bias makes the definition of the FP constant even more critical. 

**Figure 5:**
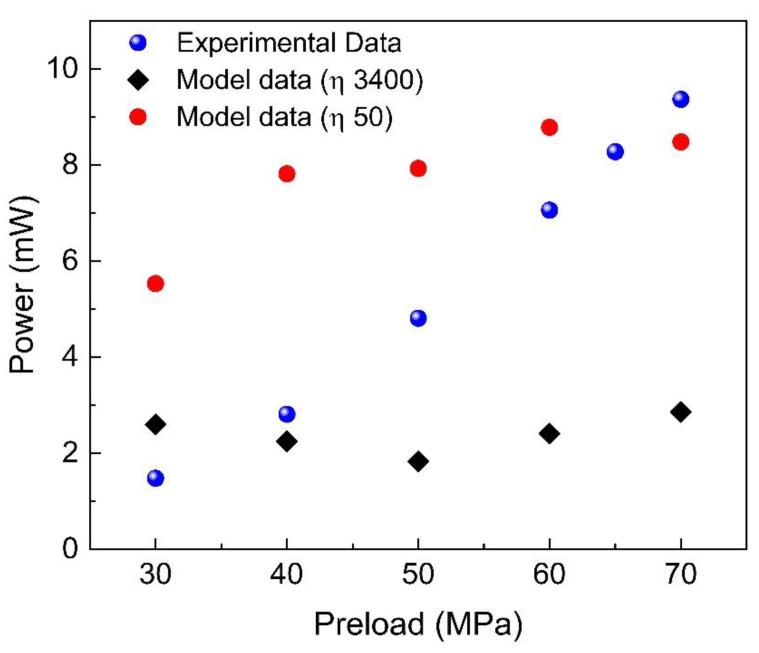
Comparison of the output power given by the model data (red circles and black diamonds) and the experimental data (blue spheres) for different values of mechanical preload.

The main consequence of the strong dependence of the Fe–Ga magnetic properties on the mechanical preload is that a single FP constant cannot ensure convergence for the whole range of the considered values, as shown in Figure 5. Here, the experimental data (points in blue) are compared with the results provided by simulation using two FP constants—*η* = 3400 and *η* = 50—having imposed 800 iterations. Experimental measurement points have been repeated and verified, taking into account the effect of wrong positioning evident in [30]. Besides accurate positioning, in order to obtain good measurement repeatability, a long warming-up period (approximatively 40 min) is required for each measurement point. The estimated measurement uncertainty is about ± 5%.

The first value of *η* would be suitable for values of reduced preloads (about 40 MPa), while the second one fits the behavior of higher values (about 70 MPa). However, it is evident that the use of a single FP constant fails, leading to calculated results not always in agreement with experiments. Thus, for the simulation of this material in dynamic conditions, a new identification procedure is needed. 

### 3.2. Model Tuning

**Figure 6:**
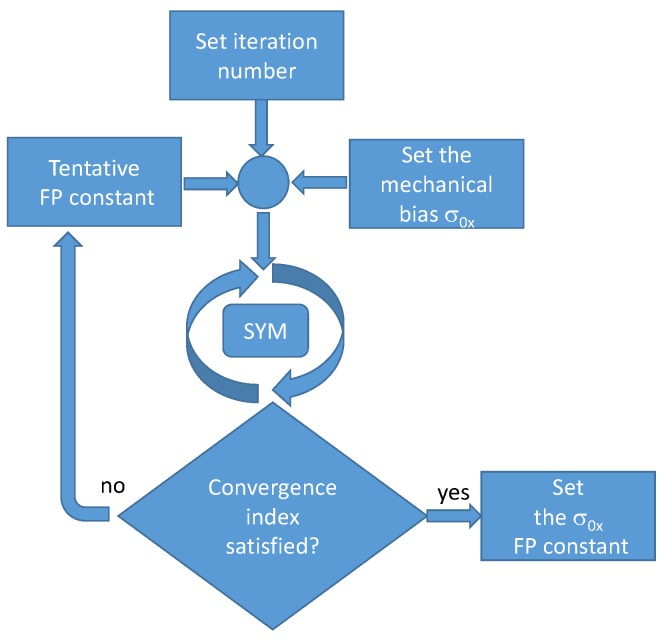
Scheme for the choice of the FP constant for a generic $\sigma_{0}{}_{x}$ mechanical preload.

The model presented in this paper is phenomenological and the first tuning is performed through identification, carried out starting from the magnetic characteristics of the material. The second tuning, which concerns the convergence of the iterative method based on the FP technique, does not require additional measurements, but considers only the value reached by the convergence index (an L2 norm of the relative difference of the solutions at two successive steps) after a stated number of iterations. For each preload value, the evaluation of the correct FP constant is obtained through a process of trial and error. Figure 6 summarizes the FP constant selection process.

Preliminary computations show that a convergence index equal to $4\times 10^{-3}$ is a good compromise between accuracy of the results and calculation times (around 10 h).

**Figure 7:**
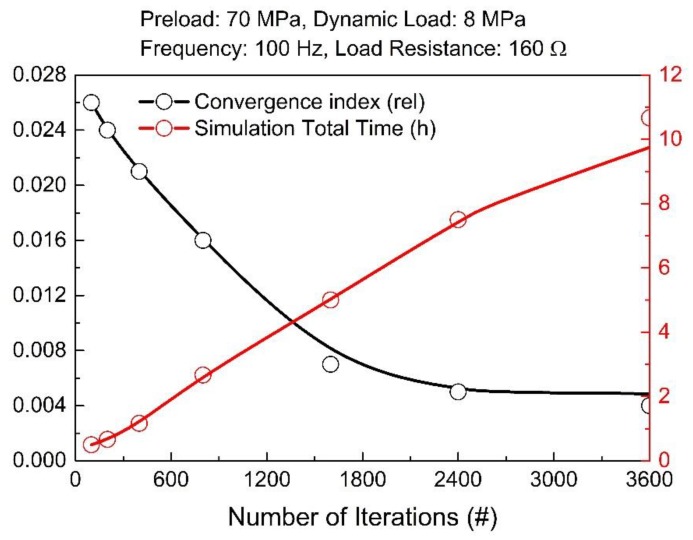
Evolution of convergence index and simulation time as a function of the number of iterations. Trend lines are just a guide for the eyes.

Figure 7 illustrates the convergence index and the simulation total time as a function of the number of iterations, for the mechanical preload value of 70 MPa.

### 3.3. Results

**Figure 8:**
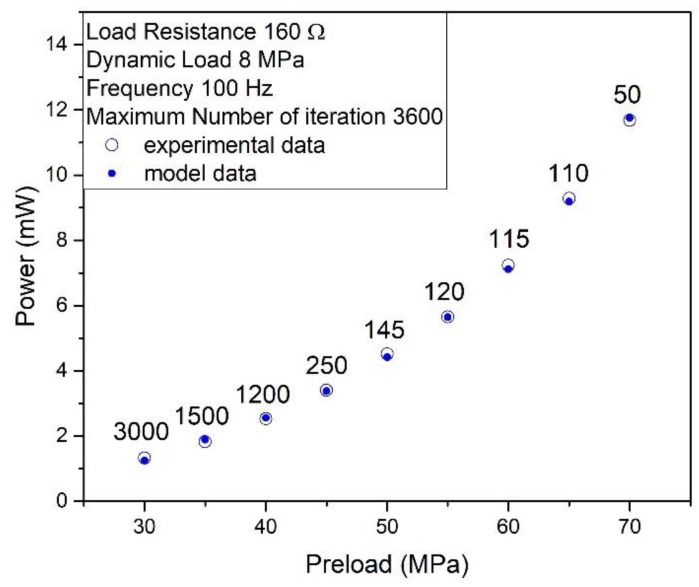
Comparison of experimental data (empty circles) and model data (solid circles) for different values of mechanical preload. The label indicates the value of the parameter *η*. Load resistance 160 Ω, dynamic load peak 8 MPa, frequency 100 Hz.

The correct identification of the model, and the correct choice of the constant FP described in Section 3.2, lead to an excellent agreement between the experimental and computational values of the output power transferred to the load, as shown in Figure 8. 

**Figure 9:**
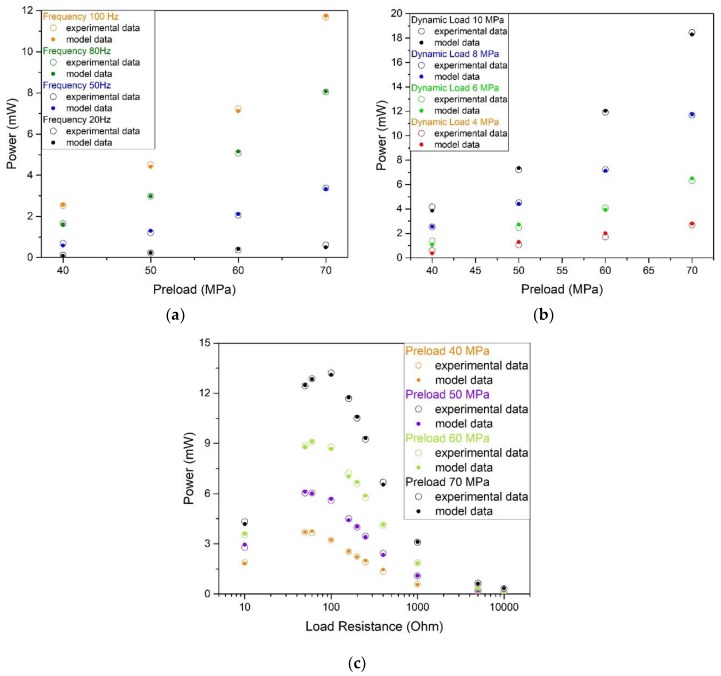
Comparison of the output power evaluated by the model, and results of experiment upon varying the following parameters: (**a**) Value of the dynamic load (from 4 MPa to 10 MPa), in which the output power is a function of mechanical preload; (**b**) frequency (from 20 Hz to 100 Hz), where the output power is a function of mechanical preload; (**c**) load resistance (from 10 Ω to 10 kΩ), for several values of mechanical preload.

The values of the FP constant previously identified for each mechanical preload (see described Figure 8) provide accurate output powers, varying all other parameters. Notably, in Figure 9a, the vibration frequency ranges from 20 Hz to 100 Hz; in Figure 9b, the dynamic load amplitude varies from 4 MPa to 10 MPa; finally, the load resistance varies from 10 Ω to 10 kΩ in Figure 9c. 

The direct dependence of the output power vs frequency (Figure 9a), or amplitude of the mechanical stress applied (Figure 9b), is easy to understand. The diagram of the output power vs load resistance (Figure 9c) shows a maximum load resistance in the range 80–90 Ω. The presence of a peak complies with the maximum power transfer theorem, which states that the maximum power provided by a source is obtained when external resistance is equal to the internal resistance of the source. In this case, the internal resistance includes both the coil electric resistance *R_c_* (32.6 Ω) and an equivalent resistance *R_e_* (estimated around 50 Ω), which takes into account the magnetic losses inside the rod.

The satisfactory agreement between experiments and computations, which is reached in all analysed situations, provides evidence that the FP constant values are not affected by parameters other than the mechanical preload. The influence of the magnetic bias, imposed by the permanent magnets, was not analysed.

## 4. Conclusions

A Preisach-type model is applied here to simulate the behaviour of an Fe–Ga harvester prototype. This approach, making a preliminary material identification, has been implemented in a 2D axis-symmetric finite element software. Non-linearities in the computational code are handled with the FP technique, whose convergence is heavily influenced by the mechanical bias values. The model requires a specific tuning, in order to determine the suitable values of *η_i_* for each preload $\sigma_{0}{}_{i}$. The tuning ensures a satisfactory result compared to validation measurements. However, the model results are unsatisfactory when using a unique value of the FP constant.

The model is able to provide effective results in a wide range of mechanical preloads and, with the experimental apparatus available for this investigation, the results have been validated up to 70 MPa. The model is also able to provide good results with an associated variation in frequency, vibration amplitude, or electrical load. 

The model has, therefore, proven to be a valid design tool for harvesters based on magnetostrictive materials, and will be used for the design of an optimized EH with magnetic yoke.
